# The Hospital World

**Published:** 1911-01-21

**Authors:** 


### The Hospital World.

### Personalia.

Princess Victoria of Schleswig-Holstein, Lady President of Buckinghamshire, presided at a meeting of the League of Mercy organised by Lady Susan Trueman in the Town Hall, Chesham, last week.

The Duke of Newcastle, Colonel Lord William Cecil, the Hon. Rupert Guinness, and Mr. Abram Lyle have been added to the list of Vice-Presidents of the Queen's Hospital for Children, Hackney Road, on the motion of Lord Shaftesbury. Speaking at a special meeting of Governors, of which we give an account in another column, Lord Shaftesbury said that the above four gentlemen had taken a special interest in the work of the hospital, and therefore he proposed their election as the first item on the agenda of the meeting that day.

The death of Lady Beatrice Moncreiffe, whose former husband was Lord Chesham, has caused much distress not only in the locality, but among many survivors from the South African War. As a writer in the *Times* reminds us : When the late Lord Chesham went to South Africa and took command of the Imperial Yeomanry during the Boer War, Lady Chesham followed him and did splendid work with the Imperial Yeomanry Hospital Corps at Deelfontein. She was always keenly interested in ambulance work, and was a Lady of Grace of St. John of Jerusalem, and had received the decoration of the Royal Red Cross.

### Elections and Appointments.

Lord Cathcart has accepted the chairmanship of the General Committee of University College Hospital.

The Duke of Devonshire has become president of the Children's Sanatorium for the treatment of phthisis, Holt, Norfolk.

Admiral and Lady Paget have been re-elected patrons of the Fancy Dress Ball Committee in connection with the Queenstown Hospital.

The election of the following ladies and gentlemen to the Board of Management of the Edinburgh Royal Infirmary has taken place : Reappointed, Mr. W. A. Tait, C.E., Mr. Walter Bell, Lady Susan Gilmour, and Dr. J. O. Affleck. Elected, Sir Robert Cranston, K.C.V.O., and Mrs. Alexander James.

At the usual fortnightly Committee meeting last Monday of the Essex County Hospital, Colchester, the Rev. Edward Spurrier was unanimously elected Chairman, and Mr. H. B. Dickinson, J.P., of Pebmarsh, Vice-Chairman of the Hospital Committee. Mr. Spurrier, it is interesting to remember, was the first Vice-Chairman of the institution, and his present appointment gives to him the wider scope to which his work for the institution clearly entitles him.

